# Self‐compassion, coping strategies and gender differences in psychology, counselling and psychotherapy practitioners during COVID‐19: Lessons learnt

**DOI:** 10.1002/capr.12574

**Published:** 2022-08-09

**Authors:** Jessica Keyes, Ala Yankouskaya, Constantina Panourgia

**Affiliations:** ^1^ Department of Psychology Bournemouth University Poole UK

**Keywords:** coping, COVID‐19, gender, psychology, counselling and psychotherapy practitioners, self‐compassion

## Abstract

Effective coping strategies can protect against the adverse effects of stress. This study investigated self‐compassion's ability to predict approach and avoidance coping in psychology, counselling and psychotherapy practitioners (PCPPs) during COVID‐19, and whether this differed between genders. To date, no research has investigated this relationship, despite positive associations being drawn in other populations. Three hundred and nineteen PCPPs completed an online survey including the Brief COPE and Self‐Compassion Short Form. Self‐compassion significantly predicted greater use of approach coping and reduced use of avoidance coping. The predictive ability of self‐compassion was slightly better in female practitioners. Self‐judgement arose as a prominent subscale of self‐compassion across genders, increasing both approach and avoidance coping. Implications for future research and practice are discussed, concerning barriers to self‐compassion and the requirement of therapeutic programmes and psychoeducation as a prerequisite for effective coping strategies in the PCPP population.

**Implications for practice and policy**

- Psychology, counselling and psychotherapy practitioners should consider their own stress management to safeguard their own mental health.
- In response to unpreceded events and work challenges, PCPPs are called to embrace self‐compassion practices to maintain or adopt approach coping strategies to support their well‐being.
- For female practitioners in particular, training and supervision should aim to draw on self‐kindness, common humanity and the positive functions of self‐judgement, as these aspects predicted approach coping. Moreover, psychoeducation targeting the negative aspects of self‐judgement is key for both male and female practitioners to ensure it is not used maladaptively, which could encourage avoidance coping.

## 1 INTRODUCTION

The Coronavirus (COVID‐19) pandemic has brought dramatic changes in everyday life and demanded extreme amounts of resilience globally (Chen & Bonanno, 2020). The current investigation highlights the vital role of psychology, counselling and psychotherapy practitioners (PCPPs) who played, and still play, an essential part in providing care for people whose mental health has been adversely affected by the pandemic. Despite the erroneous belief that PCPPs should know how to cope with stress (Pope & Tabachnick, 1994), evidence from the members of the British Association for Counselling and Psychotherapy (BACP) suggests that they are not immune to occupational stress (Ryan et al., 2019); the inherent stress of dealing with their patients' trauma renders them more vulnerable to work stress and burnout (Lamb & Cogan, 2016). Unsurprisingly, COVID‐19 deteriorated the public's mental health (Kar et al., 2021; Sheek‐Hussein et al., 2021) and has been classified as requiring disaster management (Sheek‐Hussein et al., 2021). Lambert and Lawson (2013) noted that disaster mental health counsellors who provided support throughout Hurricanes Katrina and Rita were at a greater risk of compassion fatigue, even if they had not been personally affected by the tragedies. However, with the increase in demand for mental health services due to COVID‐19, PCPPs have faced additional workload, a different nature of mental health needs, feeling deskilled, stress and a lack of preparation, in addition to their own COVID‐related anxieties potentially impacting their well‐being in a negative way (Brown, 2020) and, consequently, the quality of psychological treatment for service‐users (Delgadillo et al., 2018). Lambert and Lawson's (2013) research would therefore suggest that PCPPs are also at an extremely increased risk of compassion fatigue through the nature of their work combined with personal stress.

In such situations, PCPPs, as frontline workers, are expected to cope effectively to reduce the risk of burnout. Given the elevated work‐related stress, the new and long‐standing work challenges, and increased levels of trauma they have been exposed to, understanding the coping strategies that these professionals utilised during a period of unexpectedly high stress is vital for the promotion of their well‐being and the quality of their work.

Coping is a crucial aspect of stress (Montero‐Marin et al., 2014) and is defined as “constantly changing cognitive and behavioural efforts to manage specific external and/or internal demands that are appraised as taxing or exceeding the resources of the person” (Lazarus & Folkman, 1984, p. 141). Often, an individual's capability to cope with stress can be used as an indication of their well‐being, suggesting that successful coping can protect individuals from the deleterious effects of stress (Allen & Leary, 2010). Theoretically, the concept of coping is strongly linked to the transactional model of stress (Lazarus & Folkman, 1984), which posits that individual differences can determine different responses to stress. According to this model, the adoption of different coping strategies relies on a dynamic interaction between the individual and the environment. In other words, through this interaction, the individual produces an appraisal of a situation that consequently defines their choice of coping strategies and leads to various negative/positive outcomes.

Theorisation for universal categories of coping has been debated by many researchers (Skinner et al., 2003). Despite this, some broad categories are more widely used than others, namely approach (sometimes termed “active”) versus avoidant coping. This distinction illustrates an individual's orientation towards or away from a threat (Carver et al., 1989); approach strategies involve tackling a stressful situation directly, whereas avoidant strategies cause the individual to distance themselves from stress (Roth & Cohen, 1986). Throughout the literature, strong evidence suggests approach strategies are associated with positive outcomes, such as adjustment, increased well‐being and lower risk of burnout (Babore et al., 2020; Gustems‐Carnicer & Calderón, 2013). The adoption of avoidance coping is linked to perceived stress, poor health and worse mental health functioning (Boals et al., 2011; Holahan et al., 2005). For example, Kar et al. (2021) observed significant associations between avoidance coping strategies and greater levels of anxiety and depression in a sample of the general population and healthcare professionals in response to COVID‐19. This emphasises the concern surrounding the use of avoidance coping during the pandemic and highlights the need for interventions to help people better cope with stress.

Although evidence exists indicating the high incidence of stress and burnout among psychologists (McCormack et al., 2018) and counsellors (Ryan et al., 2019), and low professional quality of life in psychotherapists (Laverdière et al., 2019), there is a paucity of research investigating which coping strategies PCPPs employ when facing stressful situations. Cushway (1992), in a sample of clinical psychology trainees, found that the most popular coping mechanisms employed were active behavioural strategies such as speaking to friends or supervisors. In contrast, the use of avoidance coping was highly correlated with depression. Similarly, in a sample of practising clinical psychologists, it was reported that active behavioural coping strategies were utilised the most (Cushway & Tyler, 1994). Avoidance coping in this sample was again associated with poorer mental health outcomes. Hence, this research highlights a false perception that a profession skilled with teaching effective coping must be experts at practising it (Rupert & Dorociak, 2019). Early research on this perception indicates that psychotherapists employ different processes when helping clients overcome psychological distress compared with overcoming their own distress (Prochaska & Norcross, 1983). The authors offer plausible explanations, such as psychotherapists assuming they are healthier than clients, or simply not taking advantage of their skill set, both worrying suggestions that highlight the importance of helping PCPPs consider their own stress management to safeguard their own mental health (Gleeson, 2020). It has to be noted that existing literature on the topic is difficult to reconcile due to the lack of agreement about classification, types and assessment approaches to coping strategies. For example, a qualitative study with a sample of counsellors similarly underlined self‐care, social support and supervision as important coping strategies which prevent burnout (Hunter & Schofield, 2006). Although these findings may not be accurately extrapolated due to differences in coping strategy classifications, the study identifies key strategies counsellors use to cope with stress.

Due to the illustrated impact of COVID‐19 on PCPPs (BPS, 2020; Brown, 2020), the current investigation aimed to explore coping strategies within this population, as they play a crucial role in fighting off the negative effects of COVID‐19 that are still visible and may linger for the coming years. Although a large body of research has been dedicated to the adoption of coping strategies pre‐COVID‐19, a very limited number of studies have focused on the use of coping strategies in PCPPs during the pandemic. For example, only two studies (Litam et al., 2021; Panourgia et al., 2021) investigated the relationship between individual factors, perceived and COVID‐induced stress, resilience and coping strategies. These studies were consistent in their conclusion that coping responses to stress could not predict quality of professional life. However, neither of these studies examined factors that might predict the use of various coping strategies. As such, PCPPs' experiences of coping with crisis mental health issues remains unexplored, limiting informed support for this population.

In our effort to capture how PCPPs coped with the exacerbated mental health needs caused by COVID‐19, we could not ignore the role of self‐compassion, which is a very important element in job‐related stress, and is defined by Neff (2003) as “being touched and open to one's own suffering, not avoiding or disconnecting from it” (p. 87). Self‐compassion is a learnable skill and consists of three main components, each with a negative pole: (a) self‐kindness; extending kindness and understanding to the self rather than being self‐judgemental, (b) common humanity; observing one's experiences as a part of a larger common experience rather than isolating, and (c) mindfulness; holding negative thoughts in balance rather than overidentifying with them (Neff, 2003).

In general, self‐compassion has a positive effect on psychological well‐being (Neff et al., 2007). A meta‐analysis demonstrated a large effect size between high levels of self‐compassion and lower levels of mental health symptoms in students and therapists (MacBeth & Gumley, 2012). Moreover, Beaumont et al. (2016) observed that trainee counsellors and cognitive behavioural psychotherapists with high self‐compassion showed improved well‐being and reduced secondary traumatic stress and burnout when faced with stress. Furthermore, research indicates that counsellors who practise self‐compassion not only manage stress more effectively and prevent burnout, but also report higher levels of job satisfaction and personal growth (Patsiopoulos & Buchanan, 2011). Another study on trainee counsellors reported that self‐compassion is related to characteristics positively impacting the therapeutic session (Fulton, 2016). Explanations suggest that practising self‐compassion is not synonymous with avoidance of negative feelings or replacement of negative feelings with more positive ones, but involves embracement of negative emotions, resulting in the generation of positive ones too (Germer & Neff, 2013; Neff & Dahm, 2017).

The above research findings suggest PCPPs who display greater self‐compassion experience less stress and enhanced well‐being. Potentially, possession of this quality helps reduce stress by promoting adaptive ways of coping (Neff, 2003). Allen and Leary (2010) found self‐compassion was most strongly related to positive cognitive restructuring by aiding one's ability to think about a stressful situation in a different way. Furthermore, self‐compassion was negatively related to avoidance strategies which have shown links with poor psychological well‐being (Holahan et al., 2005). Similarly, a meta‐analysis (Ewert et al., 2021) revealed correlations between self‐compassion and coping, noting a lack of empirical studies investigating the influence of self‐compassion components on this relationship. A large majority of the above evidence arises from general population samples or healthcare workers, providing a gap in the research to investigate this relationship in PCPPs.

Another important observation in the literature is evidence demonstrating gender differences within self‐compassion and coping strategies. However, the expansive array of coping strategies makes comparisons between research findings a challenge (Skinner et al., 2003). In a sample of clinical psychology trainees, Cushway (1992) noted that female practitioners were more likely than male practitioners to report the use of active cognitive and behavioural coping strategies. Gender socialisation theory (Stockard, 2006) suggests there is greater acceptance for women to show emotion than men, who are more socialised to conceal their emotions; this could explain why they are less likely to report employing coping strategies as this may indicate there is a stressor they are struggling to manage. This theory could also explain gender differences in self‐compassion, suggesting self‐compassion is harder for men to engage with due to its soothing role, a quality arguably nurtured more in women (Rossi, 2018). Interestingly, research generally presents self‐compassion as a construct men possess more of (Neff et al., 2005; Raes, 2010; Yarnell et al., 2015). Considering that a large majority of PCPPs are women (Johnson et al., 2020), it could be inferred that an increased incidence of stress and burnout in this population (MacBeth & Gumley, 2012) is potentially linked to lower levels of self‐compassion. Therefore, investigations exploring gender differences in self‐compassion are pivotal given the above evidence and the overwhelming stress for PCPPs during these demanding times within their profession (BPS, 2020; Brown, 2020).

In summary, coping strategies have been identified as an important aspect of how we respond to stress. COVID‐19 required PCPPs to respond to their clients' increased needs when they were experiencing similar stressors related to the pandemic, subsequently compromising their well‐being (BPS, 2017); previous research on psychotherapists has shown that this increases the likelihood of inefficient services and poor clients outcomes (Delgadillo et al., 2018; Figley, 2002). Thus, understanding the way PCPPs coped during the first lockdown of the COVID‐19 pandemic will provide us with meaningful suggestions for mental health organisations and crisis management training programmes to prepare them for any future unprecedented situations. It is also well‐documented that self‐compassion is a significant quality for PCPPs, and evidence from trainee and practising psychologists, counsellors and psychotherapists suggests that it is positively linked with reduced stress (Beaumont et al., 2016; Finlay‐Jones et al., 2015) by promoting adaptive ways of coping (Neff et al., 2005). Furthermore, several studies observe a positive association between self‐compassion and approach coping strategies in the general population (Allen & Leary, 2010; Neff et al., 2005; Sirois et al., 2015). However, research has not yet explored self‐compassion as a predictor of coping strategies in PCPPs. There has been a lot of work on gender differences in self‐compassion showing men possessing small but significantly greater levels of self‐compassion than women (Yarnell et al., 2015). Studies have also tested gender differences in coping strategies, demonstrating that female psychologists report a greater use of active coping strategies than males (Cushway, 1992; Cushway & Tyler, 1994). Still, no study has examined gender specific differences between self‐compassion and coping strategies in PCPPs during times of extreme stress. This is unfortunate because identification of gender differences holds the potential for crisis mental health services to ensure support and stress‐management interventions can be tailored to practitioners' individual needs.

In light of the above evidence, it was hypothesised that, within the PCPPs sample: (a) self‐compassion will positively predict approach coping strategies, (b) self‐compassion will negatively predict avoidance coping strategies, and (c) there will be a difference in the predictive ability of self‐compassion on approach and avoidance coping between males and female practitioners.

## 2 METHOD

### 2.1 Sample and procedure

Data were collected from 409 participants via an online survey, but individuals who completed 50% or less of the survey were omitted from the sample, resulting in an analytical sample of 325 participants. From those participants, six participants were removed from our sample as they did not disclose their gender. Of those with valid data, 262 were women and 57 were men. The gender ratio of 0.22 in this study reflects the proportion of practising PCPPs who are members of the BPS (BPS, 2016) and the BACP (Brown, 2017). The mean age of participants was 53.34 years (*SD* = 11.49).

We collected data from clinical psychologists, psychologists, counsellors and psychotherapists who were practising face to face and/or online during the UK's first lockdown. The study was advertised via social media and professional bodies. Ethics approval was obtained from Bournemouth University's Research Ethics Committee. All participants signed a consent form electronically prior to participation.

### 2.2 Measures

*Coping* was assessed using a 28‐item scale, the Brief COPE questionnaire (Carver, 1997). This measure consists of 14 scales of two items each; six subscales measure approach coping strategies and six subscales measure avoidant strategies. The humour and religion subscales are not categorised as approach or avoidant coping strategies. Participants were asked to rate how much they used each coping strategy when under stress during the lockdown on a 4‐point Likert scale (1 = I have not been doing this at all, 4 = I have been doing this a lot), with higher scores indicating increased use of the corresponding coping strategy. In this study, alpha coefficient was 0.80 for the approach coping strategies subscale and 0.66 for the avoidant coping strategies subscale.

#### 2.2.1 Self‐compassion

The 12‐item Self‐Compassion Scale‐Short Form was used (SCS‐SF; Raes et al., 2011) to assess individual differences in self‐compassion by exploring how often individuals respond to feelings of inadequacy or suffering. The scale consists of six subscales, each containing two items. There are three positive subscales: self‐kindness, common humanity and mindfulness, and three negative subscales: self‐judgement, isolation and over‐identification. Participants were asked to rate the statements on a 5‐point Likert scale (1 = almost never, 5 = almost always). A total score is calculated by reverse scoring negative subscale items and computing a total mean. A score of between 1 and 2.5 reflects low self‐compassion, 2.5–3.5 suggests moderate self‐compassion and 3.5–5.0 represents high levels of self‐compassion (Neff, 2022). In this sample, Cronbach's alpha was 0.87.

### 2.3 Data analysis

We first tested the key assumptions of linear regression to determine whether residuals were normally distributed. The results of this analysis are reported in supplementary materials (Plot B; Plot C). We performed an exploratory linear regression modelling to understand whether self‐compassion can predict coping strategies. Two linear regression models were tested. In model one, we tested the predictive ability of self‐compassion on approach coping strategies in the total sample (both genders combined). Using the same sample, in model two, we tested the predictive ability of self‐compassion on avoidant coping strategies. In both models, regression coefficients were estimated using a bootstrapping approach with 5000 iterations. Bootstrapping is a method of random sampling with replacement that has been proven in hypothesis testing as a powerful approach for checking the stability of regression coefficients in cross‐sectional data (Galvao & Montes‐Rojas, 2015; Zhou et al., 2019). This method is based on generating thousands of independent bootstrap samples drawn from the data by permuting the individual points across the sample. Repeating the process a larger number of times provides the required information on the variability of the regression coefficient, since the standard error is estimated from the standard deviation of the statistics derived from the bootstrap samples (Walters & Campbell, 2004). Importantly, the bootstrapping procedure allows assessment of statistical accuracy by providing a confidence interval and standard error of means.

To inform our decision on the model, we computed a Bayes Factor approximation using the p‐value calibration introduced by Sellke et al. (2001). The Sellke et al. (2001) *p*‐value calibration (Vovk‐Sellke maximum p‐ratio denoted as VS‐MPR) is a function of the p‐value and is interpreted as the lower bound of the Bayes Factor (favouring *H1* to *H0*) (Altman & Krzywinski, 2017a, 2017b).

To test the effect of gender on the predictive ability of self‐compassion on approach and avoidance coping strategies, we performed four multiple regression analyses. In model one, we tested the predictive ability of self‐compassion on approach coping strategies using only the female data from our sample. In model two, we tested the predictive ability of self‐compassion on avoidant coping strategies using female data from our sample. Models three and four mirrored this, but instead used male data from our sample. This prompted further analyses. Therefore, we performed four multiple regressions using the subscales of self‐compassion (self‐kindness, common humanity, mindfulness, self‐judgement, isolation and over‐identification) as predictors and gender as a factor.

## 3 RESULTS

Descriptive statistics are presented in Table 1.

**TABLE 1:** Means and standard deviations for study variables sorted by participant gender

	Self‐compassion			Avoidant coping			Approach coping		
	1	2	3	1	2	3	1	2	3
*M*	3.30	3.42	3.32	20.39	19.07	20.15	34.30	32.07	33.90
*SD*	0.68	0.69	0.68	3.96	4.12	4.01	5.73	6.30	5.89

*Note*: 1 = female scores, 2 = male scores, 3 = scores for genders combined.

A linear regression model was conducted in JASP (version 0.16) to examine the predictive ability of self‐compassion on approach coping strategies in PCPPs. The following regression coefficients were estimated using a bootstrapping procedure with 5000 iteration. High levels of self‐compassion significantly predicted high frequency use of approach coping (*b* = 1.86, 95% CI [0.94, 2.81], *t* = 3.95, *p* < 0.001). Furthermore, self‐compassion accounted for 5% of the variance in approach coping, which was significant (*F*[1, 318] = 15.62, *p* < 0.001, VS‐MPR > 100). This supports hypothesis one that higher levels of self‐compassion can significantly predict greater use of approach coping.

A second linear regression model examined the predictive ability of self‐compassion on avoidance coping strategies, finding that high self‐compassion significantly predicted low frequency use of avoidance coping (*b* = −2.34, 95% CI [−2.94, −1.74], *t* = −7.70, *p* < 0.001). Self‐compassion explained 16% of the variance in avoidance coping (*F*[1, 318] = 59.27, *p* < 0.001, VS‐MPR > 100), therefore supporting hypothesis two that, inversely, lower levels of self‐compassion predict greater use of avoidance strategies.

Before testing hypothesis three, mean differences between genders were explored and are presented in Table 1. Men had slightly higher levels of self‐compassion on average (*M* = 3.42, *SD* = 0.69) than women (*M* = 3.30, *SD* = 0.68). Women scored higher than men on avoidant (females *M* = 20.39, *SD* = 3.96; males *M* = 19.07, *SD* = 4.12) and approach coping (females *M* = 34.30, *SD* = 5.73; males *M* = 32.07, *SD* = 6.30). An independent samples t‐test exhibited that these differences were statistically significant; women scored significantly higher than men on approach *t*(317) = 2.62, *p* < 0.01, *d* = 0.38; and avoidance coping *t*(317) = 2.25, *p* < 0.05, *d* = 0.33.

To investigate whether the predictive ability of self‐compassion on coping strategies differed between genders, a further multiple regression analysis was conducted. This showed that high self‐compassion scores significantly predicted greater use of approach coping strategies in women (*b* = 2.11, *t* = 4.18, *p* < 0.001), CI [1.21, 3.07], explaining 6% of the outcome variance (*R* ^2^ = 0.06), which was significant (*F*[1, 261] = 17.47, *p* < 0.001). In comparison, self‐compassion was not able to significantly predict the use of approach coping in males (*b* = 1.26, *t* = 1.04, *p* = 0.30), CI [−1.33, 3.43]. For avoidance coping, high self‐compassion scores significantly predicted low frequency use of these strategies in men (*b* = −2.19, *t* = −2.93, *p* < 0.01), CI [−3.52, −0.77] and in women (*b* = −2.32, *t* = −7.01, *p* < 0.001), CI [−2.93, −1.73]. However, self‐compassion explained slightly more variance in female practitioners (*R* ^2^ = 0.16) than male practitioners (*R* ^2^ = 0.14), although both models explained significant proportions of variance.

The individual subscales of self‐compassion were therefore explored further to investigate specificity in these outcomes. In this multiple linear regression, all self‐compassion subscales were entered into the model as independent variables. These subscale items were highly correlated, as seen in Figure S1, confirming the enter method of entry as most appropriate.

Results are displayed in Table 2. It was indicated that self‐kindness, common humanity and self‐judgement significantly predicted increased use of approach coping in females. Conversely, low levels of mindfulness and high levels of self‐judgement and over‐identification significantly predicted low frequency use of avoidance coping in women. Self‐compassion in men did not significantly predict use of approach coping strategies and, therefore, none of these subscales were significant. However, for avoidance coping in men, only self‐judgement was a significant predictor in the model.

**TABLE 2:** Multiple linear regression models for self‐compassion subscales

Model (dv)	IVs	Females			Males		
		*b*	*SE*	Adjusted *R* ^2^	*b*	*SE*	Adjusted *R* ^2^
1 (APP)	Self‐Kindness	1.26*	0.55	0.11	−0.5	1.44	0.16
	Common Humanity	1.32**	0.46		1.65	0.88	
	Mindfulness	0.48	0.54		1.6	1.5	
	Self‐judgement	1.15*	0.51		1.66	1.12	
	Isolation	−0.69	0.42		−1.71	1.32	
	Over‐identification	0.08	0.49		0.46	1.18	
2 (AVOID)	Self‐Kindness	0.4	0.36	0.22	0.55	0.88	0.27
	Common Humanity	0.38	0.30		0.74	0.54	
	Mindfulness	−0.79*	0.36		−1.03	0.91	
	Self‐judgement	0.75*	0.33		1.56*	0.68	
	Isolation	0.22	0.27		0.77	0.8	
	Over‐identification	0.92**	0.32		−0.19	0.72	

*p < 0.05; **p < 0.01; ***p < 0.001.

## 4 DISCUSSION

The study aimed to investigate the relationship between self‐compassion and coping strategies in PCPPs during the first lockdown in the UK and whether this relationship differed between men and women. It was found that more self‐compassionate practitioners were more likely to utilise approach coping strategies and less likely to adopt avoidance coping strategies; our findings also supported self‐compassion as a slightly better predictor of approach and avoidance coping use in female rather than male practitioners. Additionally, self‐judgement emerged as a prominent influence across approach and avoidance coping in both male and female practitioners.

In consistency with previous research in general populations (i.e. Ewert et al., 2021; Sirois et al., 2015), the current findings indicate that high self‐compassion could significantly predict higher frequency use of approach coping strategies in PCPPs. Research suggests self‐compassion encourages approach coping by helping individuals think about stressors in more constructive ways (Allen & Leary, 2010). Theoretically, self‐compassion can be associated with Lazarus and Folkman's (1984) Transactional Model of Stress which posits that appraisal of stressors is influenced by environmental and personal factors. In this instance, self‐compassion may be used as a personal resource, aiding the individual's perception of negative events as less threatening (Allen & Leary, 2010). This may subsequently encourage orientation towards stressors via approach coping strategies.

Furthermore, previous literature has drawn strong correlations between self‐compassion and self‐efficacy (Iskender, 2009; Souza & Hutz, 2016), suggesting increases in self‐compassion can enhance positive feelings about oneself and dispel negative tendencies to self‐criticise (Neff, 2003). Applied to coping strategies, “coping self‐efficacy” is a term defined as “the perception of one's capability for managing stressful or threatening environmental demands” (Benight et al., 1999, p. 2444). In the face of increased stress due to COVID‐19 (BPS, 2020; Brown, 2020), self‐compassion could be enhancing PCPPs' coping self‐efficacy, encouraging the individual to tackle stress actively using approach coping strategies (Sirois et al., 2015).

In addition, our findings revealed that high levels of self‐compassion significantly predict lesser use of avoidance coping strategies, consistent with literature inversely reporting low levels of self‐compassion often leading to a greater use of avoidance strategies (Allen & Leary, 2010; Leary et al., 2007; Sirois et al., 2015). Neff et al. (2005) suggest individuals lacking self‐compassion have an unbalanced perspective on stress due to a reduced ability to see their experience as part of a larger common experience; they may subsequently experience feelings of isolation and be more inclined to deny or disengage with stress via avoidance coping strategies (Allen & Leary, 2010). Additionally, less mindfulness towards negative emotions can result in an overwhelming focus on the negative aspects of a situation, increasing self‐criticism and reducing available resources to activate approach coping (Ewert et al., 2021). Avoidance coping in the face of stress has frequently been labelled as detrimental to well‐being (Holahan et al., 2005), and therefore, self‐compassion's ability to predict a reduction in these strategies should be nurtured.

Furthermore, self‐compassion was found to predict greater use of approach coping strategies only in women. Specifically, self‐kindness, common humanity and self‐judgement were significant aspects of self‐compassion linked to approach coping strategies in female practitioners. Research has observed positive associations between self‐kindness and self‐efficacy (Iskender, 2009), suggesting that remaining kind to oneself can enhance one's beliefs about capabilities to manage stressful situations, and consequently increase an individual's confidence to successfully implement approach coping strategies (Sirois et al., 2015). This finding is consistent with evidence showing that women are often more self‐critical and show themselves less self‐kindness than men (Smith et al., 2018). Furthermore, gender socialisation theories suggest women discuss their feelings more regularly than men (Brody & Hall, 2008). Therefore, they have more opportunities to acknowledge that failure and imperfection are part of human experience (Neff, 2003), and this could encourage motivation to implement approach coping strategies.

Nonetheless, the finding that increased levels of self‐judgement predicted increased approach coping in female practitioners was unexpected. Literature suggests a self‐critical attitude elicits rumination, self‐blame and a reduction in feelings of self‐worth (Neff, 2003), which contradicts the proactive, constructive nature of approach coping (Allen & Leary, 2010). Therefore, adopting an approach coping style could prove challenging in the face of such negative emotions. However, this unpredicted finding could be explained by the multifaceted nature of self‐judgement; some researchers argue that self‐judgement can serve several functions, such as sustaining standards and self‐correcting by focusing on the negative impact of behaviours (Castilho et al., 2015). For example, in a sample of female psychology students, Gilbert et al. (2004) observed that self‐criticism which arose from internalised standards demonstrated a significantly higher correlation with the inadequate self rather than the hated self. This implies that individuals were able to self‐criticise for not meeting their own standards, but this was separate from maladaptive self‐criticism, such as hating the self. Therefore, the current finding is perhaps not extremely unusual; it is very likely that female practitioners were engaged in self‐judgement to evaluate their ability as professionals to cope with these challenges and motivate themselves to solve problems.

It was further revealed that high self‐judgement, low mindfulness and high over‐identification predicted avoidance coping in female practitioners. Self‐judgement in this instance could encompass more contempt for the self (Gilbert et al., 2004), damaging coping self‐efficacy and leading to the use of avoidance strategies. Low mindfulness in women has been observed in other research; notably, Sabir et al. (2018) recognised that female doctors exhibited lower mindfulness than their male counterparts. Research suggests that low mindfulness may be more present in women due to their susceptibility to increasing stress levels making it harder to focus on nonjudgementally observing thoughts and feelings (Wang & Chopel, 2017). Reflecting on the current study's findings, it can be argued that similar processes are present in female PCPPs. Reduced levels of mindfulness can manifest as a refusal to accept a situation, leading an individual to suppress it from conscious awareness (Hayes et al., 1996). Avoidance coping strategies, such as denial, may therefore be adopted if female PCPPs lack the ability to acknowledge their thoughts and feelings about stress. Also, high levels of over‐identification can mask an individual's alternative thought processes and make their ability to respond differently to a situation ineffective (Neff, 2003). As demonstrated by the current findings, greater over‐identification in female practitioners could be rendering them unable to embrace objective perspectives on their stress, resulting in adoption of avoidance coping strategies. In the face of such extreme stress for PCPPs (BPS, 2020; Brown, 2020), feeling overwhelmed may increase the likelihood of resorting to avoidance coping strategies, such as denial or mental disengagement, to cope with stress (Allen & Leary, 2010).

Self‐compassion did not significantly predict approach coping strategies in male practitioners, a primarily concerning finding due to the positive outcomes associated with approach coping, such as increased well‐being and reduced stress (Gustems‐Carnicer & Calderón, 2013). This finding could be explained by the gender socialisation theory (Rossi, 2018), which posits that men are more socialised to conceal emotions, potentially making it appear as though they are coping well in stressful situations. Furthermore, the American Psychological Society (2012) notes that men are more likely than women to say they do nothing to manage their stress. This supports the notion that men may resist admitting using approach coping strategies, as it indicates there may be a problem they are struggling to overcome. However, in our investigation, self‐compassion did significantly predict a reduction in avoidance strategies in male practitioners, suggesting it is still important in reducing the negative effects of avoidance coping (Holahan et al., 2005).

Interestingly, of all self‐compassion subscales, only high self‐judgement was driving the reduction in avoidance coping for male practitioners, making it a significant predictive aspect of self‐compassion for both approach and avoidance coping in male and female practitioners. Self‐judgement also stands out in previous research; Dreisoerner et al. (2020) explored whether training one aspect of self‐compassion would improve other aspects. They discovered training in common humanity and mindfulness reduced their negative pair and increased overall self‐compassion; however, self‐kindness training did not improve self‐kindness, nor did it have any effect on self‐judgement. This paints self‐judgement as a sturdy aspect of self‐compassion, one which may be difficult to challenge. For PCPPs, this is concerning as self‐judgement has also been associated with compassion fatigue and burnout (Durkin et al., 2016).

However, in dispute of the SCS‐SF (Raes et al., 2011) used to assess self‐compassion in this study, Muris et al. (2016) noted that the association between negative subscales of self‐compassion and negative outcomes was three to five times larger than the association between positive subscales and positive outcomes. The authors argue reverse scored negative subscale items should not be included in the overall self‐compassion score because they magnify the negative. This provides some reassurance that the prevalence of self‐judgement in our sample could be a reflection of the SCF‐SF; however, this should be explored further.

The present findings provide practical implications for the use of self‐compassion in clinical settings. Wise et al. (2012) argue that, although self‐compassion is not an ethical obligation in clinical practice, competence is. This study demonstrates that self‐compassion can predict greater approach and less avoidance coping, potentially contributing to improved competence. Therefore, it is imperative that self‐compassion is included throughout training, supervision, and clinical practice for practitioners, to ensure they remain competent when faced with extreme stress.

Previous research in clinical settings has shown that self‐compassion can be acquired through psychoeducation and therapeutic programmes (Newsome et al., 2012). Our study's findings contribute to the continued requirement and development of such interventions. For female practitioners in particular, these interventions should aim to draw on self‐kindness, common humanity and the positive functions of self‐judgement, as these aspects predicted approach coping. Moreover, psychoeducation targeting the negative aspects of self‐judgement is key for both men and women to ensure it is not used maladaptively, which could encourage avoidance coping.

Although this investigation observed significant findings in a sample only demonstrating moderate levels of self‐compassion, it still implies the presence of barriers to developing high levels of self‐compassion. Gilbert et al. (2014) identified negative beliefs people hold about self‐compassion, namely that it leads to self‐indulgence, complacency, and irresponsibility. Furthermore, endorsing negative beliefs leads to lower intentions to respond self‐compassionately to events (Chwyl et al., 2020). A reduction in self‐compassion could be detrimental to PCPPs' choice of coping, as suggested by current findings, affecting subsequent well‐being (Holahan et al., 2005). Therefore, it is crucial that training and professional development programmes are developed to also dispel these negative beliefs and nurture self‐compassion.

The current investigation's findings may also benefit the practice of supervision and provide significant insight into how supervision can be tailored to increase positive outcomes for PCPPs. Supervision, an important part of counselling and psychotherapy practice and an imperative suggestion by the BACP (2021) and BPS (BPS, 2017), entails using the services of another professional in the same field and provides space for evaluation and consultation of PCPPs' personal stressors in order to maintain good standards of practice (Creaner, 2013; Inskipp & Proctor, 2001). Demonstrative of the practical implications of supervision, counsellors and psychotherapists across the literature report greater self‐awareness, improved skills, and benefits to the therapeutic relationship (Wheeler & Richards, 2007). Therefore, we should consider the applications of using supervision to cultivate self‐compassion based on the current investigation's findings of a positive association with approach coping strategies during a time of extreme stress (Chen & Bonanno, 2020). Neff (2022) expresses that we cannot offer ourselves compassion if we do not acknowledge our pain, advising that cultivation of self‐compassion involves discussion of one's own empathetic pain. Subsequently, the supervision space offers a perfect opportunity for potentially challenging self‐reflection which may increase a supervisee's self‐compassion. In addition, Coaston (2019) suggests that experiencing a supervisor being self‐compassionate towards themselves may aid the development of a supervisee's compassionate internal voice. Therefore, supervisors should use the present findings to assess and develop their own self‐compassion to help increase their supervisee's self‐compassion. Practical interventions set out to increase self‐compassion are well established, such as compassion focussed therapy (Gilbert, 2014) or compassion cultivation training (Jazaieri et al., 2014). However, in conjunction with the present research findings that greater self‐compassion and approach coping strategies are positively associated, future empirical research could focus on ways to format these interventions into supervision practice and whether the implementation of these activities during supervision impacts PCPPs' self‐compassion.

Despite the above important implications, the current investigation has some limitations. We should acknowledge that there is little agreement regarding the categorisation of coping strategies (Dubow & Rubinlicht, 2011; Stanisławski, 2019). Skinner et al. (2003) reviewed a wide range of coping measures across 100 articles, of which no two included the same coping classifications. Applications of different coping categories across studies therefore hinders the consolidation and strength of conclusions. A further limitation arises from the analysis in the current study only allowing for descriptive comparisons between the predictive ability of two regression models. To amplify these findings, research should advance these statistics to observe whether differences are statistically significant. Additionally, causality cannot be inferred from the present study due to its cross‐sectional design. Longitudinal data are needed to exclude other possibilities about the direction of the identified relations. Finally, we merely relied on self‐reports and the possibility of common source bias must be noted.

Despite these limitations, it is important not to lose sight of this study's strengths. Findings are based on data collected during the first COVID‐19 lockdown in the UK, allowing us to accurately capture PCPPs' experiences and, therefore, add knowledge to existing findings on disaster and crisis counselling (i.e. Pow & Cashwell, 2017). These findings are particularly relevant because the compounding mental health issues caused by COVID‐19 are still present and may continue into the foreseeable future. Besides, coping with stressful situations defined by uncertainty, a decreased sense of safety and lack of control are common elements of other world crises. In other words, the implications of this study's findings are informative for the design of psychologists, counsellors and psychotherapists' training on any crises management. Most importantly, this study adds to the knowledge about the relationship between self‐compassion and coping in the face of extreme stress by revealing that even moderate levels of self‐compassion can significantly predict increased use of approach coping strategies and decreased use of avoidance strategies. Based on this finding, in response to unpreceded events and work challenges, PCPPs are called to embrace self‐compassion practices to maintain or adopt approach coping strategies to support their well‐being. It was also observed that self‐compassion is slightly better at making these predictions in female practitioners. This is an important aspect of our findings considering that psychologists in the UK predominantly consist of female practitioners, as was represented in our sample (BPS, 2020; Brown, 2017).

Exploration of self‐compassion subscales provided insight into the specificity of certain aspects, such as self‐judgement, in both male and female practitioners, suggesting that psychoeducation programmes, support and supervision for PCPPs during times of national crises should specifically target this aspect of self‐compassion to ensure it is enhancing the use of approach, rather than avoidance, coping strategies. Future research should extend these findings by investigating time trajectories and examining other variables such as the effect of gender identity, age, and years of experience in the relationship between self‐compassion and coping strategies in PCPPs.

## CONFLICT OF INTEREST

The authors declare no potential conflicts of interest with respect to the research, authorship and/or publication of this article.

## DISCLOSURE STATEMENT

There is no financial interest or benefit from the direct applications of this research.

## ETHICAL APPROVAL

The study was performed in line with the principles of the Declaration of Helsinki and in accordance with the BPS Ethics code of conduct. Approval was granted by the Ethics Committee of Bournemouth University. Informed consent was obtained from all participants included in the study.

## Supporting information


Figure S1

## Data Availability

Pending acceptance for publication, all of the anonymised data files will be automatically uploaded to Bournemouth University Online Research Data repository.
