# Supplementary material for: Self‐compassion, coping strategies and gender differences in psychology, counselling and psychotherapy practitioners during COVID‐19: Lessons learnt
Source: Couns Psychother Res. 2022 Aug 9:10.1002/capr.12574. Online ahead of print. doi: 10.1002/capr.12574 (PMC9537790; doi:10.1002/capr.12574)
Supplement: Supplementary file 1 — Figure S1 [file CAPR-9999-0-s001.docx]

**Supplementary Materials**

**Self-Compassion, Coping Strategies and Gender Differences in Psychology, Counselling and Psychotherapy Practitioners During COVID-19: Lessons Learnt**

First note, the Shapiro-Wilk test was used to check normality which concluded a normal distribution across approach coping scores *W*(318) = .99, *p* = .08, however normality was violated for self-compassion scores *W*(318) = .98, *p* < .001 and avoidant coping scores *W*(318) = .98, *p* = 0.001. Central theorem theory states that when a sample size exceeds 100 or more observations, violation of normality is not an issue (Altman & Bland, 1995; Ghasemi & Zahediasl, 2012). Furthermore, the skewness and kurtosis values for all variables were within the acceptable range which demonstrates that the data for these variables is very close to a normal distribution. On this basis, the decision was made to continue the analysis using a parametric test. All assumptions for multiple linear regressions were checked and met.

**Figure 1**


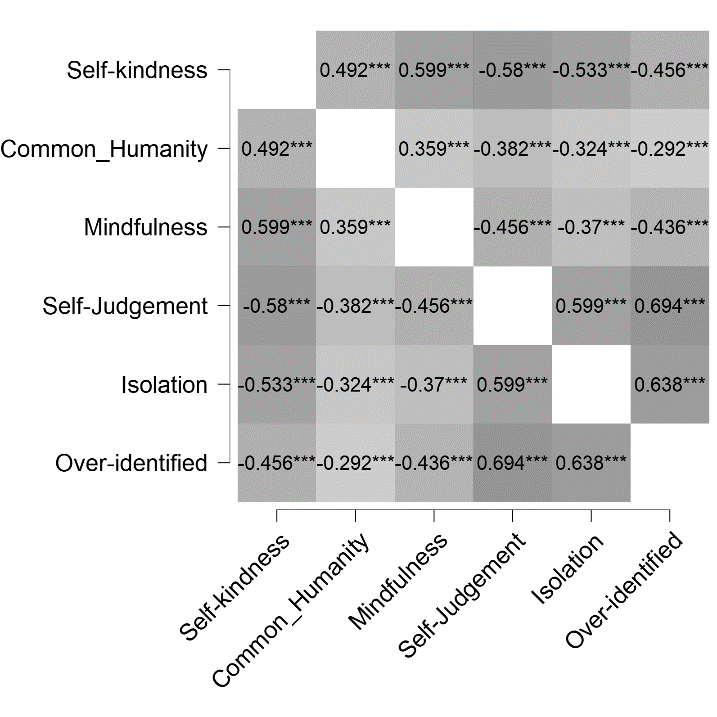
*Correlation Heat Map for Self-Compassion Sub-Scales*

*Note.* Values in squares represent Pearson correlation coefficient.

* *p* < .05, ** *p* < .01, *** *p* < .001,

**References**

Altman, D. G., & Bland, J. M. (1995). Statistics notes: the normal distribution. *British Medical Journal, 310*(6975), 298. <https://doi.org/10.1136/bmj.310.6975.298>

Ghasemi, A., & Zahediasl, S. (2012). Normality tests for statistical analysis: a guide for non-statisticians. *International Journal of Endocrinology and Metabolism, 10*(2), 486-489. <https://doi.org/10.5812/ijem.3505>
